# Identification of *Leishmania* Isolates From Healing and Nonhealing Cutaneous Leishmaniasis Patients Using Internal Transcribed Spacer Region PCR

**DOI:** 10.5812/jjm.9529

**Published:** 2014-04-01

**Authors:** Sepideh Tolouei, Seyed Hossein Hejazi, Kamran Ghaedi, Seyed Javad Hasheminia

**Affiliations:** 1 Department of Parasitology and Mycology, School of Medicine, Isfahan University of Medical Sciences, Isfahan, IR Iran; 2 Department of Biology, School of Sciences, University of Isfahan, Isfahan, IR Iran; 3 Department of Immunology, School of Medicine, Isfahan University of Medical Sciences, Isfahan, IR Iran

**Keywords:** *Leishmania major*, cutaneous Leishmaniasis, Wound Healing

## Abstract

**Background:**

Cutaneous Leishmaniasis (CL) is a parasitic disease in most parts of Iran, especially in the Isfahan province. The most common form of CL is a self-healing lesion but in rare situations, infection might develop to non-healing forms. Clinical symptoms and treatment process might be influenced by several agents such as host immune response and parasite strains. In this study, the isolates which caused healing and nonhealing forms of CL in Isfahan were characterized by internal transcribed spacer (ITS) polymerase chain reaction (PCR) technique.

**Objectives:**

The aim of this study was to identify *Leishmania* species isolated from healing and non-healing CLs using PCR method.

**Patients and Methods:**

Thirty patients resident in Isfahan province, with healing or non-healing form of CL were entered into this study. After DNA extraction, the identification of *Leishmania* isolates was done by ITS1-PCR method.

**Results:**

*Leishmania major* was found as the predominant species (100%) in both healing and non-healing forms of CL.

**Conclusions:**

It seems that there is no difference between *Leishmania* species in healing and non-healing forms of CL. In order to recognize the reason of long lasting lesions in non-healing patients, the study about parasite strains and immune factors at the molecular level mostly in nonhealing patient is recommended.

## 1. Background

Leishmaniasis is a widespread infection caused by different species of *Leishmania* that are transmitted by sandflies (1, 2). The disease divided into three forms: cutaneous leishmaniasis (CL), mucocutaneous leishmaniasis (MCL), and visceral leishmaniasis (VL) (3). In the past decade, the number of infection cases in endemic areas has increased sharply. Additionally, because of migration, travels and coinfections with HIV, it is spread to the nonendemic areas of the world (4-6). CL is a common form of disease caused by a complex of *Leishmania major*, *L. tropica*, *L. mexicania* and, *L. aethiopica* in many parts of the world (3). The disease also is endemic in various regions of Iran with a high incidence rate (7). In Iran the disease prevalence is high in some provinces such as Isfahan (8, 9).

The most common form of CL is a self-healing lesion which heals in less than one year. However a rare outcome of infection might develop to a nonhealing form of disease which lasts for several years and does not respond to various types of chemotherapies. In humans, clinical symptoms and healing process might be influenced by several agents. Thus, in order to recognize the reason of long lasting nonhealing process, the study on the type of immune response and parasite strains is very important (10). On the other hand identification of the *Leishmania* species is essential for evaluating and prescribing appropriate treatment. 

Diagnosis of chronic or nonhealing form of leishmaniasis due to its varied symptoms, manifestations and limited number of parasites in the lesion are difficult. The classic laboratory diagnosis methods, such as microscopic examination of stained-Giemsa slides, and cultivation of parasite has limited sensitivity. In addition, these techniques cannot identify the species of *Leishmania* parasite. For improving the detection and characterization of *Leishmania* spp, in the past decade, a few number of polymerase chain reaction (PCR) assays with high sensitivity were used to amplify different sequences (11-13). 

Among these sequences, internal transcribed spacer 1 (ITS1) is easy to amplify even in small quantities of DNA (11). ITS1 gene seems to be less conserved and has high degree of variation even between closely related species (14). Hence it has been used to analyze phylogenetic relationships between several organisms such as *Leishmania* (14-16). Although there are indications that Isfahan has been a major endemic area of ZCL in Iran (17), no sufficient data were available about nonhealing form of the disease in this province.

## 2. Objectives

The aim of this study was to identify *Leishmania* species isolated from healing and non-healing CL patients using PCR method.

## 3. Patients and Methods

### 3.1. Study Population

This cross-sectional study was carried out on patients who were clinically suspected to CL, and referred to the Skin Disease and Research Center of Sedigheh Tahereh Isfahan, Iran. The volunteers completed information form including name, age, gender, address, and location of ulcer on the body, data and place of acquiring the disease, previous travel history and auto immune diseases. Informed consent was also obtained from all the patients and the study was approved by Ethical Committee of Isfahan University of Medical Sciences. The patients were divided into two groups. Fifteen parasitologically proven CL patients with healing form of lesion with a onset of less than six months and no history of CL treatment and fifteen parasitologically proven CL patients with nonhealing form of lesions with duration of lesion more than one year and history of at least two courses of Glucantime treatment were included in this study. Subjects with a history of immuo-deficient or chronic disease were excluded from the study.

Diagnosis was done based on observation of *Leishmania* using Giemsa stained smears and/or growth of promastigotes in NNN culture. Identification of *Leishmania* isolated was done using PCR method.

### 3.2. Samples Preparation

Debris removed from the lesions by normal saline. The samples were obtained from edge of active lesions by scarping the skin. The amount of scraped material smeared on a microscopic slide, and stained with Giemsa. Another part of sample was transferred to Brain Heart Infusion broth (BHI), overlayed of biphasic Novy-Nicole-Macneal (NNN). The culture was incubated at 25˚C and the growth of *Leishmania* promastigotes was checked every three days. After the proliferation of promastigotes, they were transferred to RPMI 1640 medium (Gibco, Germany) supplemented with 10% FCS (Sigma, Germany) and antibiotics. Promastigote in logarithmic phase was stored at -70˚C with 8% glycerol for further manipulation.

### 3.3. DNA Extraction

DNA extraction was performed on promastigotes obtained from RPMI 1640 medium. 3-4 × 10^6^ cultured promastigotes were harvested by centrifugation (2,000 rpm) at 4˚C for 10 minutes and washed three times in cold sterile PBS (pH = 7.2). DNA was extracted by High Pure PCR template preparation Kit (Roche, Germany) according to manufacturer’s instruction. Finally DNA was resuspended in 200 μL of elution buffer, 1 μL of DNA was used as template in the PCR reaction. Quality and quantity of extracted DNA was analyzed by agarose gel electrophoresis (1%) and spectrophotometry, respectively.

### 3.4. PCR Assay

ITS1 region and the two primers (design by ALEL ID 6 software), Leish F (5´-CAA CAC GCC GCC TCC TCT CT-3´) and Leish R (5' -CCT CTC TTT TTT CNC TGT GC-3') all were used to diagnose and identify the *Leishmania* species. Amplification reaction was performed in a volume of 25 μL containing, 2.5 μL of 10X PCR buffer, 0.2 mM dNTP, 1.5 mM MgCl_2_, 5 pmol of each primers and 0.25U Taq DNA polymerase. The reaction was performed in a Thermocycler (Corbett) with the following procedure: initial denaturation at 95˚C for 5 minutes followed by 25 cycles containing denaturation at 95˚C for 30 seconds, annealing at 58˚C for 30 seconds and extension at 72˚C for 30 seconds and the end post extension phase at 72˚C for 5 minutes. Finally 5 μL of PCR products with the loading buffer were loaded in 1.5% agarose gel containing 0.5mg/mL ethidium bromide. The samples were run at 5 V/cm along with a gene molecular marker of 50 bp. The products were visualized by UV light using a transilluminator. As a control the primer was evaluated with *Leishmania* standard species including *L. major* (MHOM/IR/75/ER), and *L. tropica* (MHOM/IR/o4/Mash10).

## 4. Results

**Figure 1.**
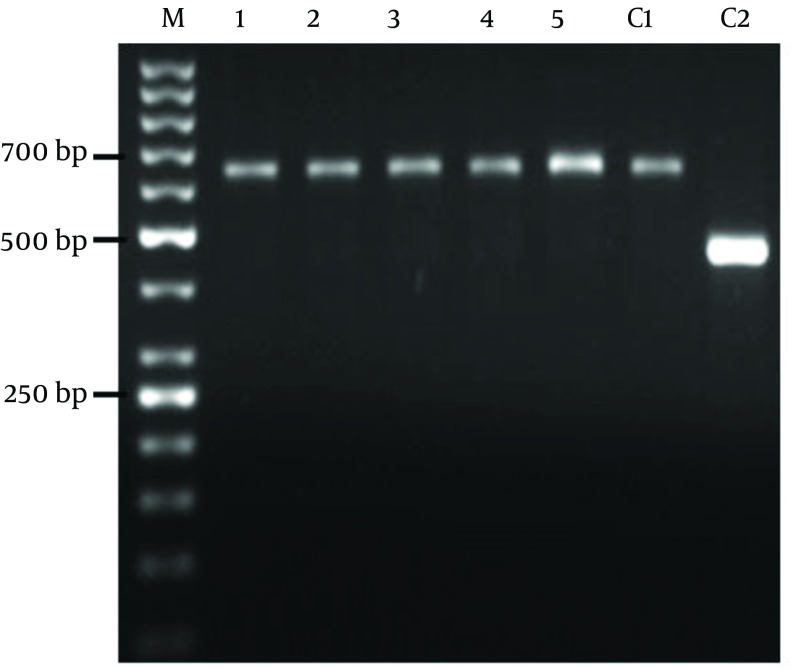
PCR Product of the Internal Transcribed Spacer1 (ITS1) Region of Genomic DNA Samples Collected From Healing and Nonhealing Lesions DNA from *L. major* (MRHO/IR/75/ER) was used as control C1, *L. tropica *(MHOM/IR/o4/Mash10) was used as control C2. Lanes 1, 2 and 3 are samples collected from healing patients and lane 4 and 5 were obtained from non-healing individuals. M, is a 50 bp DNA ladder (Fermentas)

The patient demographic characters including gender, age, number of lesions, ulcers duration, and distribution of the skin lesions on the bodies are shown in (Table 1). In this study, all the samples collected from suspected cases of healing and nonhealing forms of CL were observed by light micrsocopy (100X) and parasite culture. By microscopic examination 80% of healing and 50% of non-healing cases were positive. After cultivation of samples 100% of those in each group were positive (Table 2). Electrophoresis patterns from each isolates in two groups compared with standard strains of *L. major* and *L. tropica*. In this study, a single 625 bp band for *L. major *and 485 bp band for *L. tropica* were observed. 100% of samples in healing and non-healing form of CL identified as* L. major *(Figure 1).

**Table 1.** Major Characteristics of Patient's with Cutaneous Leishmaniasis

	Healing Cases	Non-healing Cases
**Age, y**	34 ± 10.7	38 ± 12
**Age Range**	(18-55)	(22-60)
**Gender**		
Male	12	13
Female	3	2
**Average duration of lesion, mo**	2.1 ± 1	17.3 ± 4.7
**Number of lesion**	3.1 ± 1.4	2.5 ± 1.08
**Hand and arm, %**	38.9	37.3
**Foots, % **	30.1	32.9
**Track, % **	19.4	20.5
**Face and neck, %**	11.6	9.3

**Table 2.** Diagnostic Methods Used for Detection of Healing and Nonhealing Forms of CL

Study Groups	Microscopic Examination, %	Culture, %	PCR, %	
			***L. major***	***L. tropica***
**Healing **	80	100	100	-
**Non-healing**	50	100	100	-

## 5. Discussion

The most common presentation of CL is the self-healing form which often heals in less than one year (3). However, rare cases of infection, which last for several years are known as non-healing forms of CL. Non healing form often caused by *L. tropica*, but there are few reports of* L. major* nonhealing infection (18). It seems that clinical manifestation varies based on the type of immune response generated and the species, virulence and polymorphism of *Leishmania* (19-21). Several data show the differences between immune factors in healing and nonhealing forms of disease (22, 23). The identification of parasite species is basically essential for appropriate chemotherapy and study of host immune system.

Traditionally, direct detection of parasites is possible by microscopic examination or by cultivation. In this study it was revealed that *Leishmania* parasite was less detected by microscopic observation (50%) in non-healing form of CL compared to the healing form of CL. Due to low number of *Leishmania* in chronic or nonhealing form of disease diagnosis of parasite by smearing is not suitable. On the other hand these methods cannot identify the species of *Leishmania* parasite. PCR-based methods have provided the ability to diagnose and identify *Leishmania* species (24-26). Many different PCR targets, including, splice leader mini-exon (SLME), genomic or kinetoplast DNA (kDNA) can be used as a basis for evaluating new molecular diagnostic assays for leishmaniasis (11, 27). Diagnostic PCR assays using the internal transcribed spacer 1 (ITS1) region of the rRNA genes which only need 40 to 200 copies have been shown to be sensitive methods for detecting cutaneous (CL) (11).

 In the present study molecular method was used in order to identify *Leishmania* species in healing and non-healing form of CL. Both species, *L. major* and *L. tropica* are reported as etiologic agents of CL in Isfahan (28). However in this study it was appeared that *L. major* was caused human non-healing CL. There are several studies that showed *L. major* is a main agent of healing form of CL in Isfahan (7, 17). Hejazi et al. have shown *L. major* is main agent of active form of CL in Isfahan by monoclonal antibody and PCR method (28). In other study using ITS1-PCR method also showed L. major as the predominant species of healing form of CL in Isfahan region (29). Doudi et al. was reported that among 209 isolated cases in Isfahan 205 was *L. major*. They also showed that the most prevalent genotypes related to isolates of Isfahan were LmA geneotype (96.2%) (30).

Dabirzadeh et al. also detected the genetic polymorphism of *L. major* in Isfahan, and showed that strain A was more frequent than the other strains (31). We used to ITS1-PCR method for identification and differentiation of *Leishmania* species in healing and non-healing form of disease. Our molecular result indicated that L. major was the main species in both groups of CL in Isfahan region and 625 bp band were observed after gene amplification in ethidium bromide-stained gels for all the patients. Altogether it seems that there is no difference between *Leishmania* species in these two groups but more satisfactory results would be achieved with more non healing cases. Conclusively in order to recognize the reason of long lasting of the lesion in non-healing patients, study about parasite strains and its polymorphism in the level of SNP (single nucleotide polymorphism) is very important. On the other hand study of immune factors on the molecular level is recommended.
